# Metabolomics Analysis and Modeling Suggest a Lysophosphocholines-PAF Receptor Interaction in Fibromyalgia

**DOI:** 10.1371/journal.pone.0107626

**Published:** 2014-09-19

**Authors:** Pierluigi Caboni, Barbara Liori, Amit Kumar, Maria Laura Santoru, Shailendra Asthana, Enrico Pieroni, Antonella Fais, Benedetta Era, Enrico Cacace, Valeria Ruggiero, Luigi Atzori

**Affiliations:** 1 Department of Life and Environmental Sciences, University of Cagliari, Cagliari, Italy; 2 Department of Biomedical Sciences, University of Cagliari, Cagliari, Italy; 3 Biomedicine Department, CRS4, Cagliari, Italy; 4 Department of Medical Sciences “Mario Aresu”, University of Cagliari, Cagliari, Italy; Imperial College London, United Kingdom

## Abstract

Fibromyalgia Syndrome (FMS) is a chronic disease characterized by widespread pain, and difficult to diagnose and treat. We analyzed the plasma metabolic profile of patients with FMS by using a metabolomics approach combining Liquid Chromatography-Quadrupole-Time Of Flight/Mass Spectrometry (LC-Q-TOF/MS) with multivariate statistical analysis, aiming to discriminate patients and controls. LC-Q-TOF/MS analysis of plasma (FMS patients: n = 22 and controls: n = 21) identified many lipid compounds, mainly lysophosphocholines (lysoPCs), phosphocholines and ceramides. Multivariate statistical analysis was performed to identify the discriminating metabolites. A protein docking and molecular dynamic (MD) study was then performed, using the most discriminating lysoPCs, to validate the binding to Platelet Activating Factor (1-alkyl-2-acetyl*-sn-*glycero-3-phosphocholine, PAF) Receptor (PAFr). Discriminating metabolites between FMS patients and controls were identified as 1-tetradecanoyl*-sn*-glycero-3-phosphocholine [PC(14∶0/0∶0)] and 1-hexadecanoyl*-sn*-glycero-3-phosphocholine [PC(16∶0/0∶0)]. MD and docking indicate that the ligands investigated have similar potentialities to activate the PAFr receptor. The application of a metabolomic approach discriminated FMS patients from controls, with an over-representation of PC(14∶0/0∶0) and PC(16∶0/0∶0) compounds in the metabolic profiles. These results and the modeling of metabolite-PAFr interaction, allowed us to hypothesize that lipids oxidative fragmentation might generate lysoPCs in abundance, that in turn will act as PAF-like bioactivators. Overall results suggest disease biomarkers and potential therapeutical targets for FMS.

## Introduction

FMS is a complex illness to diagnose and treat, with several related syndromes. Collectively FMS is characterized by widespread pain associated with a variety of other signs and symptoms, such as restless sleep, tiredness, fatigue, anxiety, depression and disturbances in bowel function. In the past, the most widely adopted diagnostic criteria were those of the American College of Rheumatology (ACR) [1] that defined FMS as a condition characterized by widespread pain and by the presence of at least 11 out of 18 specific tender points on physical examination. In 2010, the ACR published diagnostic criteria for FMS that encompass the chronic widespread pain, fatigue, unrefreshing sleep, cognitive symptoms, such as confusion, forgetfulness, inability to concentrate, and somatic symptoms, such as myalgia, paresthesia, dizziness, constipation, chest pain, fever, etc, previously considered the hallmarks of this condition [2]. Due to lack of knowledge about the pathogenesis, researchers studying fibromyalgia strive to identify objective, measurable biomarkers that may help to discover susceptible individuals, thus facilitating disease diagnosis and management. Despite this effort, nowadays no laboratory test has been validated for the diagnosis, which remains basically clinical. This is one of the most important reasons for the long diagnostic delay in FMS. Recently, oxidative stress with lipid peroxidation induced by reactive oxygen species has been proposed both as a relevant event in the pathogenesis of FMS and as a factor related to clinical symptoms [3]–[5]. When phospholipids are oxidized by radicals, several different types of oxidative products are formed. These include phospholipids containing fatty acid oxidation products, lysophospholipids and fragmentation products of fatty acid oxidation. Oestvang et al [6] suggest that lysophosphocoline might act through the PAFr leading to a wide range of cell events, including: arachidonic acid release, phospholipase D activity and cytosolic phospholipase A2 stimulation.

In this context, metabolomics analysis could be a valuable help. Metabolomics is an effective post-genomic research tool, that has been applied to many disciplines, including the study of human diseases, food control and plant physiology. In clinical context, metabolomics has recently been used to study relevant rheumatic diseases [7], [8]. The metabolomics approach includes various separation and detection techniques, such as gas and liquid chromatography coupled to mass spectrometry (GC-MS, LC-MS), nuclear magnetic resonance (NMR) and capillary electrophoresis/mass spectrometry (CE-MS). LC-Q-TOF/MS provides high resolution and reproducibility and is frequently used to identify compounds through the analysis of fragmentation patterns [9]. The application of metabolomics represents a major advance towards understanding human metabolic components and it allows application in the diagnosis of several diseases [10]. In order to gain knowledge in the pathogenesis of fibromyalgia syndrome, in this study we investigated the metabolic profile of patients with the fibromyalgic syndrome. We focused mainly on plasma lipidomic analysis by LC-Q-TOF/MS and patient-discriminating metabolites such as PC(14∶0/0∶0) and PC(16∶0/0∶0). These metabolites, having a PAF-like structure, were also evaluated by docking them to PAFr and modeling the complex dynamics.

## Materials and Methods

### Patients

A group of 22 females affected by FMS and 21 controls was enrolled for this study. The study was approved by the ethical committee of the University Hospital of Cagliari and written informed consent was obtained from each woman before inclusion. Diagnosis was based on a history of widespread pain, defined as bilateral, upper and lower body, as well as in the spine, and the presence of excessive tenderness on applying pressure to 11 out of 18 specific muscle–tendon sites (Tender Points), according to the ACR classification criteria [1]. All the patients also fulfilled the new ACR diagnostic criteria [2]. The presence of a major clinical condition other than fibromyalgia was excluded by physical examination and routine blood and urine screening. All FMS patients were examined by a qualified rheumatologist and were interviewed by the examining physician using the Fibromyalgia Impact Questionnaire (FIQ) [11]. Clinical monitoring included the 100 mm non anchored horizontal Visual Analogue Scale (VAS) for pain (patient and medical VAS pain). Blood samples were simultaneously collected. After centrifugation, plasma samples were stored at -80°C until use.

### Metabolomic analysis

#### Reagents

All chemicals used in this study were of analytical grade. Methanol and chloroform were used as extraction solvents. 1-nonadecanoyl*-sn*-glycero-3-phosphocholine was used as internal standard and acetonitrile was used as a solvent Sigma (St. Louis, MO, USA).

#### Plasma sample preparation

100 µL plasma samples were extracted by slight modification of the Folch procedure [12]. The first step consisted in the addition of 250 µL of methanol and 125 µL of chloroform containing nonadecanoyl*-sn*-glycero-3-phosphocholine at 10 mg/L. Samples were then kept for 1 h at room temperature. 380 µL of chloroform and 90 µL KCl 0.2 M were subsequently added and samples were centrifuged for 10 minutes at 13,200 rpm. The aqueous phase was separated from the organic layer and both phases were dried overnight under a gentle nitrogen stream. The organic layer was resuspended with 250 µL of ACN and samples were filtered with Acrodisc Syringe Filters with 0.45 µm PTFE Membrane (SIGMA, St. Louis, MO, USA).

#### LC-Q-TOF/MS analysis

The organic layers were analyzed by reverse-phase LC on an Agilent 1200 series LC system fitted with a microchip technology using an Agilent Zorbax 300 SB-C18 5 µm, 43 mm×75 µm (Agilent, Santa Clara, CA). The LC conditions were as follows: flow rate: 0.4 µL/min; solvent A: 0.1% formic acid in water; solvent B: acetonitrile; and gradient was from 5% to 100% B over 10 min. The samples (1 µL) were then analyzed by ESI in positive mode using an Agilent 6520 Time of Flight (TOF) MS. Mass spectral data were acquired in the range *m/z* of 100–1,500 with an acquisition rate of 1.35 spectra/s, averaging 10,000 transients. The source parameters were adjusted as follows: drying gas temperature 250°C, drying gas flow rate 5 L/min, nebulizer pressure 45 psi, and fragmentor voltage 150 V. On the basis of the original acquisition files, we performed a pre-processing step with MetAlign software used for automated baseline correction and alignment of all extracted mass peaks across all samples. Results were stored as CSV file. ESI/QTOF MS data were then analyzed using the molecular feature extraction algorithm of the MassHunter Workstation software (version B 03.01 Qualitative Analysis, Agilent Technologies, Santa Clara, CA, USA). The molecular feature extraction algorithm took all ions into account exceeding 1000 counts with a charge state equal to one. Blank runs showed a maximum of 10 features with the intensity threshold at 1000 counts. Isotope grouping was based on the common organic molecules model (See Table S1 for LC-MS raw data)

#### Statistical analysis

Principal components analysis (PCA) of LC-MS Q-TOF analysis data was performed using SIMCA software package (version 13.0, Umetrics, Umea, Sweden). PCA is a data clustering and visualization method that is useful to extract groupings within multivariate data. Data is represented in *n* dimensional space, where n, the number of variables, is reduced into a few principal components (PC's), which describe the maximum variation within the data. The PC's can be displayed in a graphical fashion as a “scores” plot. This plot is useful for observing any groupings in the data set and, in addition, for highlighting outliers, that may be due to errors in sample preparation or instrumentation parameters. Coefficients by which the original variables must be multiplied to obtain the PC's are called “loadings”. The numerical value of a loading of a given variable on a PC shows how much the variable has in common with that component, that is how much the variable contributes to the selected PC. The PCA showing a degree of intrinsic clustering (unsupervised) suggests that a PLS-DA of the same data should produce robust classification models. Partial least squares (PLS) regression is a well-known method to find the relationship between predictor variables X and dependent variables y. In a PLS model, not only the variance of X, but also the covariance between X and y is taken into account. Therefore, the central point of PLS is to find latent variables in the feature space that have a maximum covariance with y. PLS-DA is a variant of PLS to improve the separation between classes using a categorical response variable y. Model performance was evaluated using the squared correlation coefficient R^2^ (goodness of fit) and the cross-validated correlation coefficient Q^2^ (goodness of prediction), both of which vary between 0 and 1. In details, R^2^ provides an indication of how much of the variation within a data set can be explained by the various components of the model, while Q^2^ indicates how accurately the data can be predicted. A good prediction model is achieved when Q^2^>0.5, and an excellent prediction for Q^2^>0.9. The contribution plot showing the influence of each process variable to the statistic was calculated (SIMCA 13.0, Umetrics, Umea, Sweden). A high contribution of a process variable usually indicates a role of this specific variable. The misclassification table, showing the proportion of correct classification, and summarizes how well the selected model classifies the observations into the known classes.

### Modeling of PAFr and ligands

The starting three dimensional structure of the PAFr was obtained from the homology model realized by previous authors [13]. The structures of the two lysoPC ligands – PC(14∶0/0∶0) and PC(16∶0/0∶0) – and PAF compound were modeled using the web-server CORINA [14]. All the three ligands have a polar head and a long lipid chain, with PC(14∶0/0∶0) being the smallest ligand among them. The structures of the ligands were subjected to geometry optimization using the Hartree-Fock basis set HF-6-31G* and the Gaussian03 package [15]. The charges and the force field parameters for the ligand were evaluated following the standard AMBER protocol [16]. The optimized structures of the ligands were then subjected to docking analysis using standard protocol in Autodock [17] software package. Before MD simulations, each ligand-PAFr complex was embedded in a POPC lipid bilayer, mimicking the cell membrane environment, and subsequently inserted in a water box [18]. After an initial relaxation and equilibration run of 3 ns, a production run for a simulation length of 50 ns for the three systems was performed. MD simulations were performed using NAMD software with AMBER 99 force field parameters [19]. MD analysis concerned in particular the evaluation of: 1) Root Mean Square Deviation (RMSD) of ligand heavy atoms, allowing to measure the stability and identify the configurations of the ligand inside the PAFr; 2) durable H-bond interaction (at least 10% of the simulation time), providing relevant contribution to the binding energy; 3) binding free energies for the complexes, calculated by using SIETRAJ software [20], allowing us to quantitatively estimate binding affinity.

## Results

### LC-Q-TOF/MS analysis

**Figure 1:**
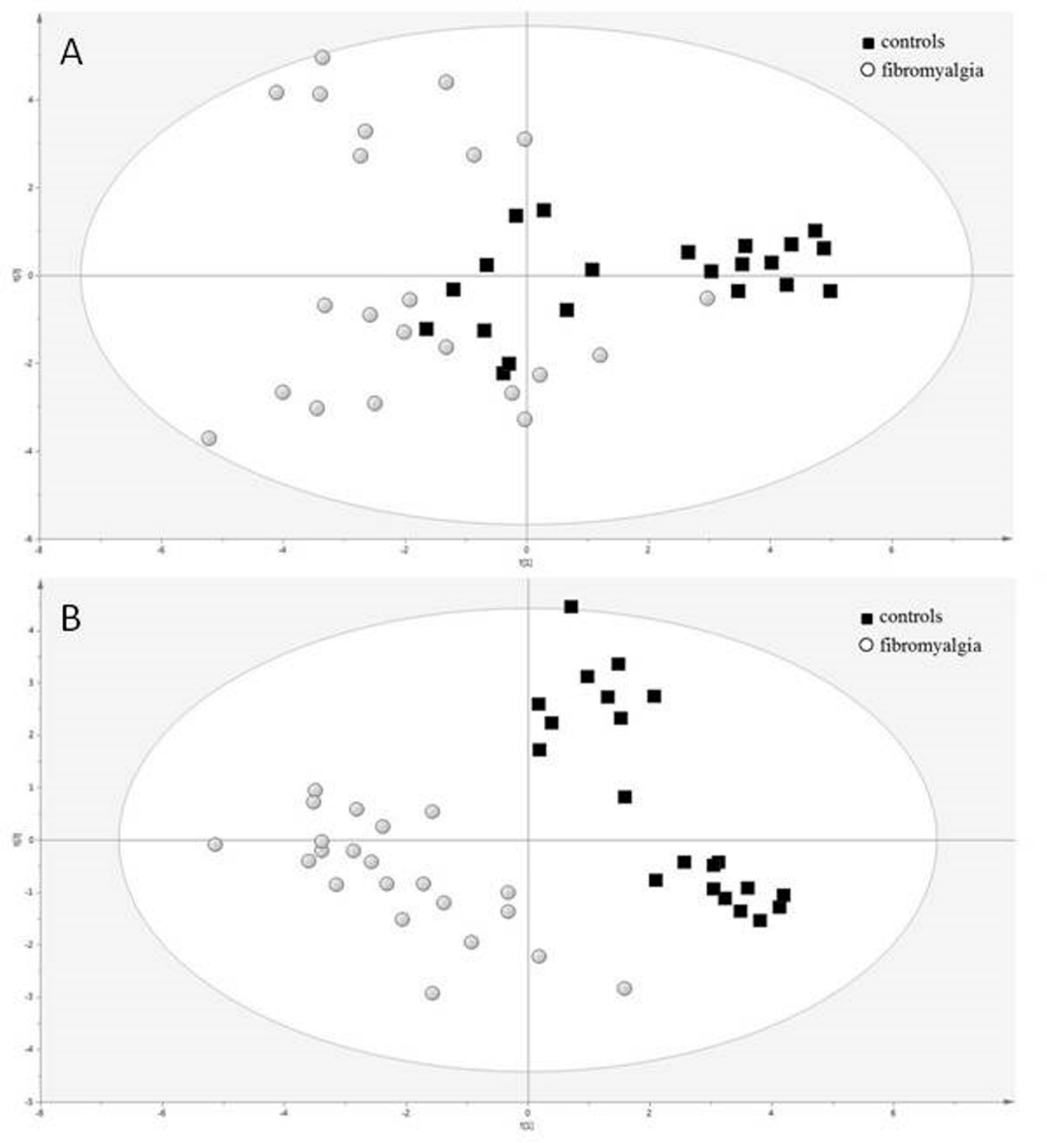
PCA (A) and PLS-DA (B) of plasma analysis from patients with FMS vs controls. PLSA-DA values were: R^2^X = 0,345 R^2^Y = 0.901 Q^2^ = 0,806 p-value = 1.1×10−12.

**Figure 2:**
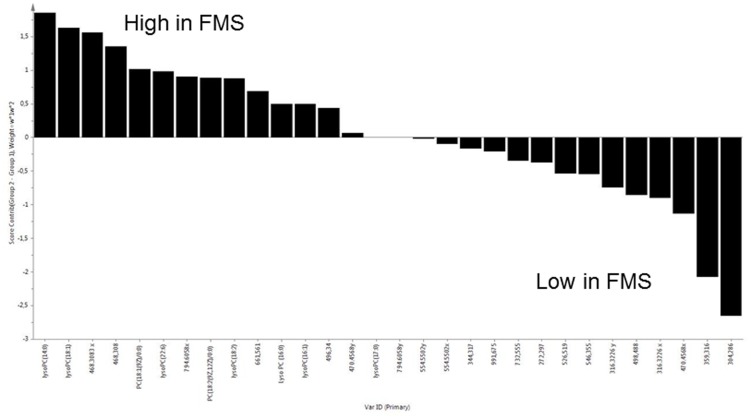
Contribution plot of plasma analysis from patients with FMS vs controls.

Demographic characteristics and clinical parameters of FMS and Control subjects are reported in Table 1.LC-Q-TOF/MS analysis of the organic layer of plasma identified lipid compounds, mainly lysoPCs, phosphocholines and ceramides. The unsupervised PCA analysis of the metabolites identified by LC-Q-TOF/MS didn't show any outlier or intrinsic distribution, therefore all subjects were used for the following supervised PLS-DA analysis (Fig. 1). Quality assessment parameters of the resulting model are R^2^X = 0.345, R^2^Y = 0.901, Q^2^ = 0.806 and p-value = 1.1×10^−12^. PLS-DA analysis of the identified lipids showed a relevant and significant difference between the two groups, fibromyalgia *vs* controls (Fig.1). In order to describe the individual contribution of the identified metabolites a Contribution Plot has been created by using the different loadings (Fig.2). By loadings plot analysis the most relevant molecules discriminating the two classes were: PC(14∶0/0∶0), PC(16∶0/0∶0), 1-(9E-hexadecenoyl)*-sn*-glycero-3-phosphocholine, 1-heptadecanoyl*-sn*-glycero-3-phosphocholine, 1-(9Z-octadecenoyl)*-sn*-glycero-3-phosphocholine, 1-(2E,4E-octadecadienoyl)*-sn*-glycero-3-phosphocholine and 1-(4Z,7Z,10Z,13Z,16Z,19Z-docosahexaenoyl)*-sn*-glycero-3-phosphocholine. When the plasma non polar compounds were analyzed by GC/MS we were not able to discriminate FMS from controls (data not shown). The ability of the model to correctly classify the individual subjects was evaluated by the misclassification table tool available in SIMCA. Misclassification analysis was able to correctly classify all the subjects under investigation (Table 2). Among the most discriminant metabolites, PC(14∶0/0∶0) and PC(16∶0/0∶0) have a PAF-like structure, so the ability of these lysoPCs to interact with PAFr was further investigated by MD.

**Table 1:** Demographic characteristics and clinical parameters of FMS and Controls.

	FMS (n = 22)	CONTROLS (n = 21)
Age [years, mean (SE), range]	52 (3) (27–72)	50 (2) (27–67)
Menopause	n = 15	n = 9
Body Mass Index (m^2^/kg)(mean ± SE)	26 (1)	23 (0.6)
Pain score patient (VAS mm) (mean ± SE)	57 (7)	-
Pain score physician (VAS mm) (mean ± SE)	40 (4)	-
FIQ total score (2–100) (mean ± SE)	61±21	-
*Associated clinical distresses*		
Tension type headache	n = 16	-
Irritable colon	n = 16	-
Oto vestibule syndrome	n = 13	-
Paraesthesia	n = 14	-
Sleep disturbance	n = 14	-
Dysmenorrhea	n = 4	-
Hemicrania	n = 8	-
Urethral syndrome	n = 8	-
*Medication*		
No therapy	n = 7	n = 21
Tricyclic*	n = 8	-
SSRI**	n = 2	-
SSNRIs	n = 5	-

* Amitriptyline <15 mg/die).

** Selective Serotonin Reuptake Inhibitors (Citalopram 20 mg/die).

***Selective Serotonin Noradrenaline Reuptake Inhibitors (Duloxetin 30 mg/die).

**Table 2:** Misclassification table showing the proportion of correctly classified observations in FMS patients and control.

Class	Members	Correct (%)	FMS	CONTROLS	No class
FMS	22	100	22	0	0
Control	21	100	0	21	0
No class	0	0	0	0	0
Total	43	100	22	21	0

Fishers probability: 9,50E-13.

### Docking and Molecular Dynamics Analysis

**Figure 3:**
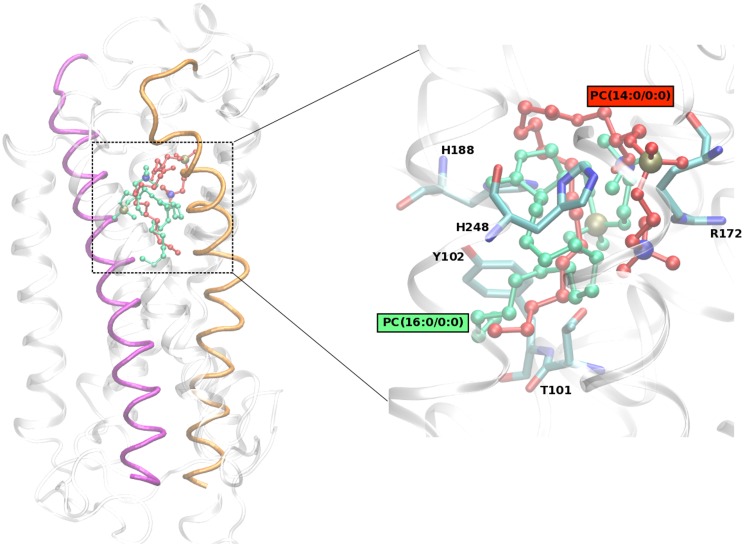
Best binding pose obtained for the ligands (i) PC(14∶0/0∶0) in green and (ii) PC(16∶0/0∶0) in red inside PAF receptor. The receptor is shown using cartoon representation with helix 3 in pink and helix 6 in orange. The ligands are shown using ball and stick representation.

The initial docking analysis suggested the most relevant binding sites for all the lysoPC ligands to be located inside the PAFr. Furthermore, docking results indicated that PAFr displays a preferential binding towards PC(14∶0/0∶0) with respect to the other ligands (Table 3). Free energies obtained by docking also show that the ligand PC(14∶0/0∶0) displays for PAFr a binding energy very similar to the physiological PAF ligand PC(O-16∶0,2∶0) (Table 3). The complex structures obtained from docking analysis were then subjected to MD simulations. We analyzed in detail the physicochemical properties of the ligand-PAFr binding dynamics, as reported in the next paragraphs. The final docking pose of the ligands inside PAFR is shown in Fig. 3 (Molecular movies of ligand PC docked inside PAF receptor during MD simulations are shown in Movie S1 and S2). Ligand RMSD inside the PAFr was evaluated (Table 3). Analyzing in details the RMSD distribution histograms, a moderate difference was observed between the PC(14∶0/0∶0) and PC(16∶0/0∶0) ligands, both showing essentially a single configuration peak, corresponding to a unique and well defined chemical binding configuration. Regarding the PAF ligand, the RMSD peak value is shifted to the left by ∼2 Å, with respect to the other two lysoPC ligands. In summary, PC(16∶0/0∶0) and PC(14∶0/0∶0) ligands appear to be slightly more flexible than PAF, but overall the three ligands display similar binding configurations inside the receptor. We evaluated H-bond interactions between the ligands and the PAFr. Notably, a conserved and durable H-bond interaction involving single residue R172 in Loop E2 and the two lysoPC ligands was observed. PC(14∶0/0∶0) ligand is also involved in H-bond interactions with residues from Helix 2, Helix 6 and Loop E2, while PC(16∶0/0∶0) is involved with residues from Helix 3, Helix 5, Helix 6 and Loop E2. Finally, the free energies were estimated. These values are coherent with the previous H-bond analysis and importantly confirm that PAF, PC(14∶0/0∶0) and PC(16∶0/0∶0) ligands display a similar binding affinity for PAFr (Table 3).

**Table 3:** Binding free energies for the three ligand-receptor complexes after docking and after MD simulations.

Ligands	ΔG (kcal/mol) MD	ΔG (kcal/mol) Docking	RMSD (Å)
PC(14∶0/0∶0)	−11.0±0.4	−7,1	1.8±0.4
PC(16∶0/0∶0)	−11.2±0.4	−5.1	1.9±0.4
PC(O-16∶0,2∶0)	−11.1±0.4	−7.0	1.3±0.4

RMSD value in the last column refers to the average value of ligand during the whole MD simulation time.

## Discussion

To the best of our knowledge, no metabolomic study has been reported on plasma of FMS patients, although a metabolomic approach might be useful to better understand the pathogenesis of this disorder and identify potential biomarkers. This is the first study showing a distinct metabolomic profiles of FMS patients and controls. In particular LC-Q-TOF/MS analyses revealed an higher level of lysoPCs in FMS patients with respect to controls. We focused on the LCMS determination of lyso PC because human serum levels of lyso PC is about several hundred nanomoles per milliliter, while PAF is generally present at very low concentrations in many biological specimens. Lipid oxidative fragmentation generates lysoPCs, implicating that they could be a source of PAF-like bioactivators in FMS. LysoPCs are formed in human plasma: the palmitic acid- and stearic acid-containing species are formed by the lecithin-cholesterol acyltransferase reaction, the oleic acid- and linoleic acid-containing species by the action of PLA_2_ [21]. LysoPCs containing myristic and palmitic acid may also derive from the action of PAF-acetylhydrolase on oxidation products of phosphocholine [22] consequent to LDL oxidation and/or oxidative stress. The latter process could explain our data showing an increase of plasma PC(14∶0/0∶0) and PC(16∶0/0∶0) in FMS.

Assessment of thiobarbituric acid reactive substances for the measurement of lipid peroxidation revealed values over two-fold of the normal range in FMS patients (data not shown), indicating a condition of oxidative stress. These data might be consistent with a previous study [23] that showed unexpected normal levels of uric acid despite high levels of inosine, hypoxanthine, xanthine in FMS patients. Uric acid is a powerful free radical scavenger in humans that might not be increased in FMS patients due to its consumption as a reactive oxygen species scavenger. Therefore, it is reasonable to speculate that lysoPCs might act through the PAFr mediation to activate a wide range of cell events [6]. The higher affinity of the lysoPC here investigated toward PAFr supports the hypothesis that these ligands are able to induce the PAF receptor open state, activate it and then initiate the signal transduction cascade through G-protein-PAFr coupling. In our case, through modeling and MD analysis, we observed a binding energy and configuration of PC(14∶0/0∶0) and PC(16∶0/0∶0) similar to that obtained for PAF, the PAFr physiological ligand (Table 3). The difference in binding free energies obtained with docking and with MD simulations can be due to the fact that conformational changes of the receptor are not considered in the docking protocol. Moreover, docking was performed without a priori information mainly to identify the relevant binding sites. Considering the simulation error in evaluating the binding free energies by MD, our results indicate that all the ligands here investigated have similar potentialities to activate the PAFr (Table 3). Previous experimental [24] and simulation studies [13] have suggested histidine residues located in helix 5 (His 188) and helix 6 (His 248, His 249) to be important for ligand binding. Our results confirm this observation, with His 188 involved in H-bond interactions with both PAF and PC(16∶0/0∶0), His 248 with PC(14∶0/0∶0) alone, and His 249 with PAF alone. The RMSD variation observed for helix 6 for all the three ligands is smaller with respect to what reported in MD simulations by previous authors for another PAFr antagonist [13]. Nevertheless, we noticed an identical peak for PAF and PC(16∶0/0∶0) RMSD distribution in helix 6. The other ligand, PC(14∶0/0∶0), tends to show in helix 3 higher RMSD values in the second half of the simulation time, that can be interpreted as an evidence of a conformational change upon binding, which again could be linked to receptor activation. In detail, PAF is a lipid mediator derived from cell membrane and it is implicated in a variety of physiological and pathological conditions [25]–[28]. Because of such a wide and relevant biological activity, the levels of PAF in cells and in blood are strictly regulated at very low levels. The intercellular actions of PAF are mediated through a G-protein–linked receptor, PAFr, that is expressed on the surface of a variety of cell types [29]–[31] and can be inhibited by a great variety of chemical compounds [32]. Thus, it seems evident that PAFr can induce a variety of intracellular signaling pathways evoking a wide range of biological functions [27].

Accumulating evidence indicates that the PAF/PAFr system plays a role in modulating pain signaling. For instance, some studies show that local injection of PAF into peripheral tissues, such as skin, enhances pain sensitivity in animals and humans [33], thus suggesting that PAF is an important substrate in the complex biological pathways leading to the pain. In the central nervous system, it has been reported that PAF is implicated in the induction of pain behaviors. Morita et al. [34] demonstrated that intrathecal injection of PAF produced potent tactile allodynia in mice, suggesting that PAF in the spinal cord may be a mediator of neuropathic pain following peripheral nerve injury [35]. Some authors [36] indicate that neuropathic pain may also be a component of FMS. In fact, a hallmark of neuropathic pain syndrome is tactile allodynia; for instance other Authors [37] reported that the activation of the PAFr may be a key event in the development and maintenance of tactile allodynia and production of pro-inflammatory cytokines such as TNFα and IL-1β in the rodent dorsal root ganglion.

## Conclusions

In conclusion, our results indicate, in a relatively small number of patients needing to be further validated, the capability of a metabolomics approach to identify distinct metabolic profiles for FMS patients and controls, suggesting a possible role for lysoPCs as biomarkers or a contribute to the disease phenotype by having a role in the pathogenesis of this condition. Our metabolomics results indicate a lysoPCs increase in FMS patients. Through modeling, we focused the molecular dynamics analysis of the binding capability of an endogenous ligand PAF and two exogenous-derived lysophosphocolines having a structure similar to PAF. In particular PC(14∶0/0∶0) and PC(16∶0/0∶0), may have a PAF-like function, activating PAFr and supposedly playing a role in the clinical manifestation of FMS. So, these results suggest potential new disease biomarkers and new approaches for treatment in FMS.

## Supporting Information

Table S1 **LC-Q-TOF/MS raw data from Control (n = 21) and FMS patients (n = 22).**
(XLS)

Movie S1 **Molecular movie of Ligand PC(14∶0/0∶0) docked inside PAF receptor during 50 ns of MD simulations.** The receptor is shown using cartoon in grey and the ligand using licorice representation and in red.
(MPG)

Movie S2 **Molecular movie of Ligand PC(16∶0/0∶0) docked inside PAF receptor during 50 ns of MD simulations.** The receptor is shown using cartoon in grey and the ligand using licorice representation and in green.
(MPG)
